# Improvement in the neutron beam collimation for application in boron neutron capture therapy of the head and neck region

**DOI:** 10.1038/s41598-022-17974-7

**Published:** 2022-08-12

**Authors:** Naonori Hu, Hiroki Tanaka, Ryo Kakino, Syuushi Yoshikawa, Mamoru Miyao, Kazuhiko Akita, Teruhito Aihara, Keiji Nihei, Koji Ono

**Affiliations:** 1 Kansai BNCT Medical Center, Osaka Medical and Pharmaceutical University, Takatsuki, Japan; 2 Institute for Integrated Radiation and Nuclear Science, Kyoto University, Osaka, Japan; 3 Central Department of Radiology, Osaka Medical and Pharmaceutical University Hospital, Takatsuki, Japan; 4 Department of Radiation Oncology, Osaka Medical and Pharmaceutical University Hospital, Takatsuki, Japan

**Keywords:** Applied physics, Particle physics, Translational research, Cancer therapy, Head and neck cancer, Techniques and instrumentation

## Abstract

In June 2020, the Japanese government approved boron neutron capture therapy for the treatment of head and neck cancer. The treatment is usually performed in a single fraction, with the neutron irradiation time being approximately 30–60 min. As neutrons scatter in air and loses its intensity, it is preferable to bring the patient as close to the beam port as possible to shorten the irradiation time. However, this can be a challenge, especially for patients with head and neck cancer, as the shoulders are an obstacle to a clean positioning. In this study, a novel neutron collimation system for an accelerator based neutron source was designed to allow for a more comfortable treatment, without compromising the irradiation time. Experimental measurements confirmed the simulation results and showed the new collimator can reduce the irradiation time by approximately 60% (under the same condition where the distance between the source and the patient surface was kept the same). The dose delivered to the surrounding healthy tissue was reduced with the new collimator, showing a 25% decrease in the D_50_ of the mucosal membrane. Overall, the use of the newly designed collimator will allow for a more comfortable treatment of the head and neck region, reduce the treatment time, and reduce the dose delivered to the surrounding healthy tissue.

## Introduction

Boron neutron capture therapy (BNCT) is a binary treatment modality that selectively kills cancer cells. It is based on the nuclear reaction that occurs when a thermal neutron is captured by a ^10^B nucleus, resulting in particles with high linear energy transfer (LET). These high-LET particles (alpha particle and ^7^Li nuclei) have ranges that are roughly equal to the size of a human cell. Clinical trials of BNCT that utilised ^10^B-para-boronphenylalanine (L-BPA) as the boron delivery agent have been reported for the treatment of recurrent head and neck cancers^1–4^. Historically, BNCT was performed using neutrons generated from nuclear reactors. Accelerator-based neutron sources are becoming more popular, as they offer several proven advantages over a nuclear reactor. The world’s first accelerator based neutron source for clinical BNCT was designed and developed by Sumitomo Heavy Industries, in collaboration with Kyoto University BNCT research group^5,6^. This accelerator was used in a clinical trial treating recurrent or locally advanced head and neck cancer between 2016 and 2018^7^. The same type of accelerator system was installed in September 2016 at the Kansai BNCT Medical Center of Osaka Medical and Pharmaceutical University. On March 11, 2020, the Japanese Ministry of Health, Labor and Welfare approved the BNCT system (NeuCure® System) as a novel medical device that can be manufactured and sold for clinical use, together with the dose calculation program (NeuCure® Dose Engine). The NeuCure® Dose Engine utilises the general-purpose Monte Carlo particle transport simulation code system called Particle and Heavy Ion Transport code System (PHITS) version 3.2 to simulate both the neutron and photon transport^8^. BNCT has been approved for coverage under the national health insurance system for unresectable, locally advanced, and recurring cancer of the head and neck region as of June 2020. Currently, the center has treated over 60 head and neck patients using the NeuCure® System (with the standard type of collimator) along with Steboronine® (^10^B enriched borono-L-phenylalanine) produced by STELLA PHARMA corporation.

For BNCT, it is preferable to bring the patient as close to the beam port as possible to keep the treatment time short, since neutrons scatter through the air and the intensity drops. This is important as the Steboronine® is only approved for infusion for a total of 3 h (the infusion rate for the first two hours is 200 mg/kg/h and the last hour is 100 mg/kg/h). The neutron irradiation is performed when the infusion rate is dropped to 100 mg/kg/h, so the maximum irradiation time (while the BPA is being infused) is limited to 1 h. However, bringing the patient close to the beam port is not easy. This is because the current system only has a single fixed horizontal beam line, and the patient needs to move toward the neutron beam port (unlike conventional radiotherapy, where the gantry rotates around the patient). This is troublesome, particularly for patients with cancer near the hypopharynx area, as the shoulders are an obstacle that prevents bringing the tumour area close to the beam port. Inevitably, this produces an air gap of the order of several centimeters between the beam port and the patient surface, and in some cases greater than 10 cm. This air gap increases the treatment time and an unnecessary exposure to healthy parts of the body.

This paper investigates an improvement in the beam collimator design that can be easily adapted to the current system to address the issues mentioned above.

## Material and method

### Extended collimator design

**Figure 1:**
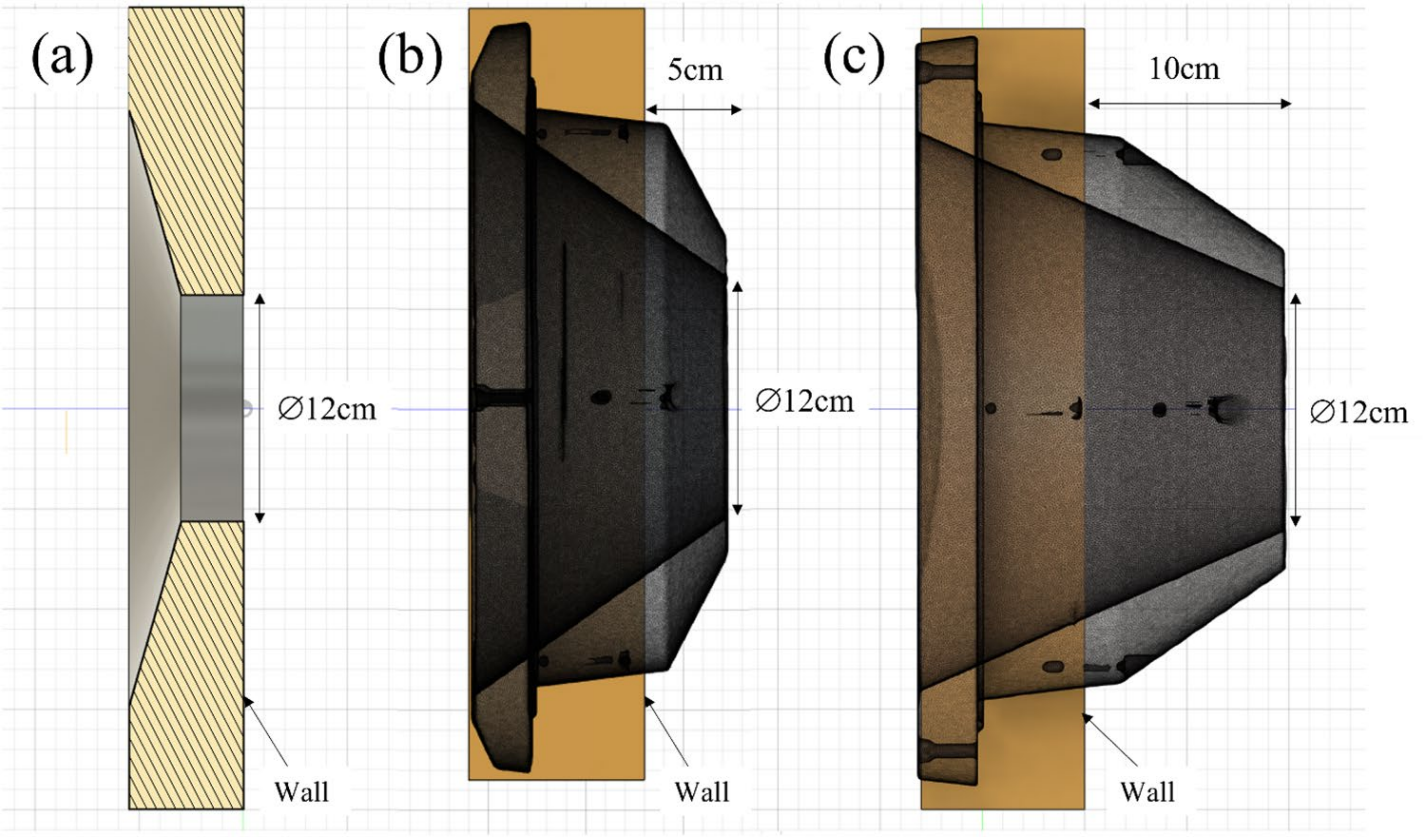
Design of the (**a**) standard collimator, (**b**) 5 cm extended collimator and (**c**) 10 cm extended collimator generated using computer aided design software. Each square block in the figure is equal to 1 × 1 cm^2^.

**Figure 2:**
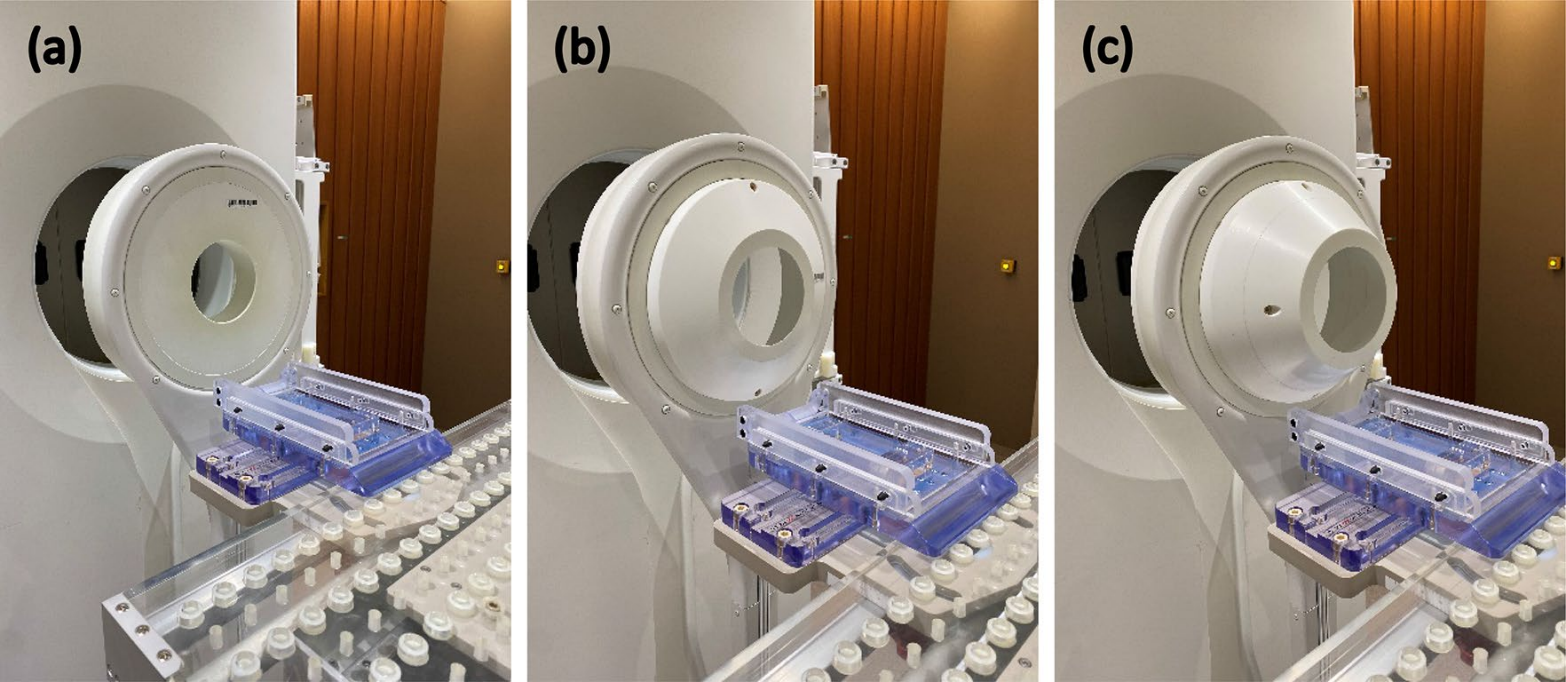
The three collimator designs. (**a**) The standard collimator, (**b**) the 5 cm extended collimator, (**c**) the 10 cm extended collimator. The beam aperture size was 12 cm diameter for all collimators.

A computer aided design software was used to design the collimator (Fig. 1). Two designs were investigated: a 5 cm and a 10 cm extension for the collimator. The diameter of the circular opening abutting the moderator (upstream) was approximately 30 cm and the beam aperture diameter was 12 cm (downstream). The collimator material was polyethylene loaded with natural LiF. The system was designed such that it would be compatible with the existing collimator system and be interchangeable to suit different clinical situations (Fig. 2).

### Beam characterisation

#### Experimental measurement—water phantom

Thermal neutron and gamma ray distribution inside a water phantom was measured using gold foil activation method and thermo-luminescent dosimeters (TLDs), respectively. Thin gold wires (diameter of 0.25 mm with a 99.95% purity, The Nilaco Corporation, Tokyo, Japan) were placed inside the water phantom (H: 28 cm, L: 21 cm, W: 21 cm with the phantom walls having a thickness of 1 cm (except for the front wall having a thickness of 2 mm)) along the central beam axis to measure the central axis thermal neutron flux and perpendicular to the beam axis to measure the off-axis thermal neutron flux. After neutron irradiation, the activated gold wires were cut into small pieces (approximately 5 mm length) and the 412 keV prompt gamma rays were measured using a high purity germanium detector. As gold reacts to both thermal and epithermal neutrons, cadmium covers were used to differentiate between the two. For the measurement of gamma ray dose rate, neutron insensitive TLDs were placed inside the water phantom to measure the depth dose distribution due to gamma radiation, along the central axis. A custom made BeO TLD enclosed in a quartz glass was used^9^. A proton charge of 0.3 C was delivered for both the thermal neutron and gamma ray measurements. Detail on the methodology of thermal neutron flux and gamma ray dose rate determination and can be found elsewhere^10^.

#### Experimental measurement—free-in-air

Gold foils and TLDs were placed free-in-air along the surface of each collimator at 1–2 cm intervals to measure the collimator leakage. The data analysis was the same as above.

### Monte Carlo simulation

#### Water phantom

The neutron and gamma ray spectrum of the NeuCure® system has been modelled and verified previously^10^. The extended collimators were modelled in detail according to the design mentioned above. The thermal neutron and gamma ray dose rate inside the water phantom was simulated using the T-Track tally. The number of primary particles run was set to obtain a relative error lower than 0.5% for the thermal neutron flux and less than 1% for the photon dose rate at a depth of 10 cm along the central beam axis.

#### Effect of an air gap on the dose distribution

The effect of an air gap between the beam exit (collimator surface) and the phantom surface on the dose distribution was investigated for the three different collimators. The dose distribution inside a 30 cm × 30 cm × 30 cm cubic phantom (density of 1 g/cm^3^ with a weight fraction of 0.1, 0.1, 0.03, 0.77 for hydrogen, carbon, nitrogen, and oxygen atom, respectively)) assuming a uniform distribution of ^10^B with a concentration of 25 µg/g was simulated. The total dose was determined by summing each of the four main dose components (boron dose ^10^B(n,α)^7^Li, nitrogen dose ^14^ N(n,p)^14^C, hydrogen dose ^1^H(n,n’)p, and the gamma ray dose originating from both the primary beam and the ^1^H(n,γ)^2^H reaction). Parameters summarised in Table 1 were used to calculate the biologically weighted dose (Gy-eq). The boron distribution inside a cell is taken into account by the Compound Biological Effectiveness (CBE), which is dependent on the boron compound type and the type of tissue being examined. Detail on the dose calculation and parameters used to calculate the biologically equivalent dose can be found elsewhere^10^. The irradiation time required to deliver a maximum dose of 12 Gy_w_ to the mucosal membrane was calculated (assuming the phantom was considered as all mucosal membrane) along with the advantage depth, which is a parameter used to evaluate the performance of a neutron beam. It is defined as the depth where the biologically weighted dose in the tumour region equals the peak value of the biologically weighted dose in the healthy tissue region (mucosal membrane). Furthermore, the off-axis dose distribution at a depth of 2 cm was calculated and the 80% and 50% isodose width was determined to investigate the effect an air gap has on the lateral beam profile.

**Table 1:** The CBE and RBE parameters used for the dose calculation.

Tissue type	CBE	RBE_N_	RBE_H_	RBE_γ_	Tissue to blood ratio
Tumour	3.8^11^	2.9	2.4	1	3.5^12^
Skin	2.5^13^	2.9	2.4	1	1
Bone	1	2.9	2.4	1	1
Brain	1.34^14^	2.9	2.4	1	1
Soft tissue	1.34^14^	2.9	2.4	1	1
Water	1	0	2.4	1	1
Air	0	0	0	0	0

### Treatment planning of a mock head and neck BNCT

#### Case 1: Cancer of the left nasopharynx

**Figure 3:**
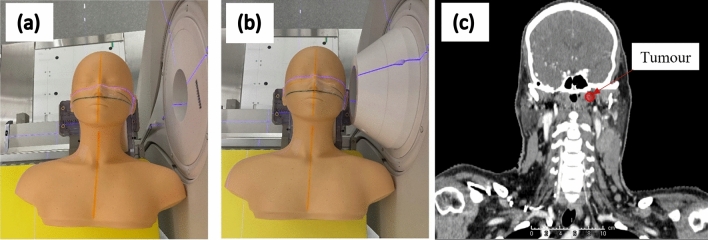
Simulation of a BNCT treatment of the left nasopharynx area (with the patient on the treatment couch) using (**a**) the standard collimator and (**b**) the 10 cm extended collimator. The coronal CT scan with the mock tumour is shown in (**c**).

A cancer of the left nasopharynx was simulated using a dummy patient dataset. A hypothetical 1 cm^3^ spherical tumour was placed below the base of the skull on the left side of the patient at a depth of approximately 6 cm from the surface. Simulation was performed using the standard collimator and the 10 cm extended collimator. The beam was simulated to enter from the left side of the patient (Fig. 3). The RT-PHITS (RadioTherapy package based on PHITS) functionality was used to convert the CT images of the dummy patient into a cubic 3 mm^3^ voxel phantom. The dose prescription was set to a maximum dose of 12 Gy_w_ to the mucosa membrane. The same parameters were used as above to calculate the biologically weighted dose. The neutron and photon flux inside each individual voxel was simulated and converted to dose using the KERMA coefficients. The simulated 3D dose distribution file was converted into a DICOM RT dose file and imported into the 3D slicer software for analysis. The dose volume histogram of the mock tumour and the healthy tissues were evaluated. All methods were performed in accordance with the relevant guidelines and regulations (e.g., Declaration of Helsinki).

#### Case 2: Cancer of the hypopharynx

**Figure 4:**
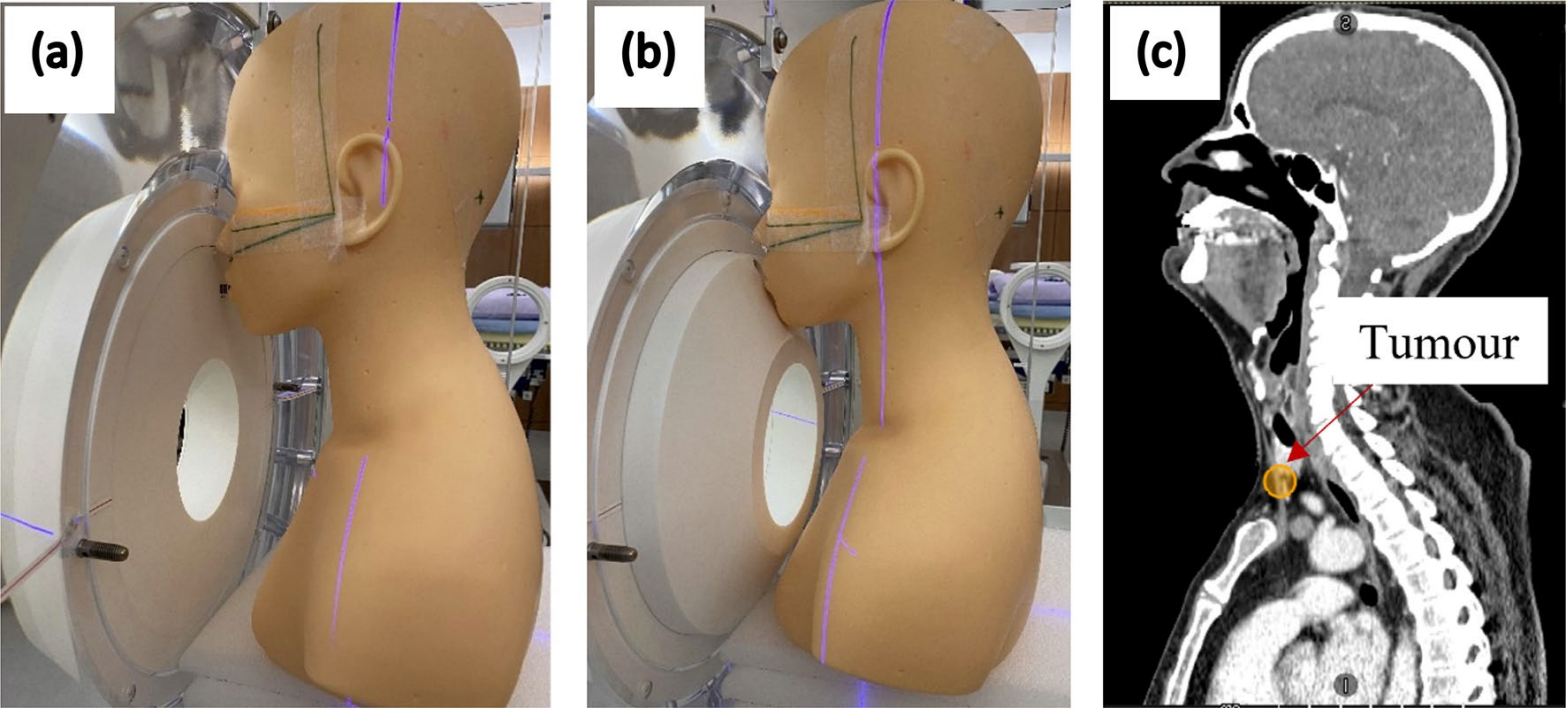
Simulation of a BNCT treatment of the hypopharynx area (with the patient in the seated treatment position) using (**a**) the standard collimator and (**b**) the 5 cm extended collimator. The sagittal CT scan with the mock tumour is shown in (**c**).

A cancer of the hypopharynx was simulated with the beam entering anteriorly. A hypothetical 1 cm^3^ spherical tumour was placed below the thyroid, in front of the esophagus, midline of the patient. Simulation was performed using both the standard collimator and the 5 cm extended collimator (Fig. 4). The same simulation parameters and calculation methods were used as above.

## Result

### Beam characterisation

**Figure 5:**
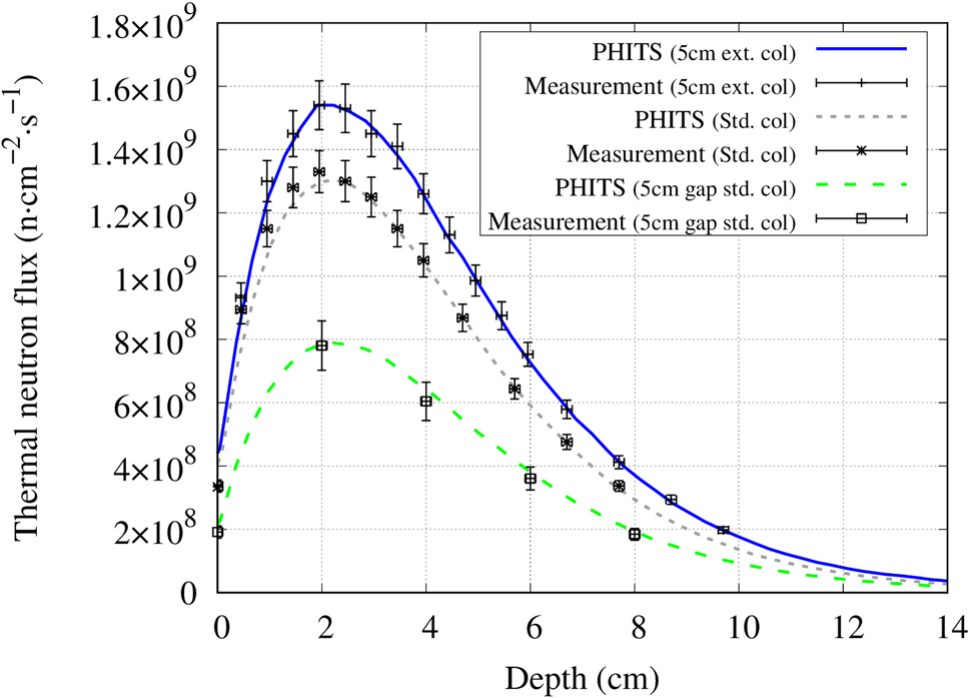
Central axis thermal neutron flux distribution inside a water phantom for the standard collimator, 5 cm extended collimator and the standard collimator with a 5 cm air gap between the collimator and the phantom.

**Figure 6:**
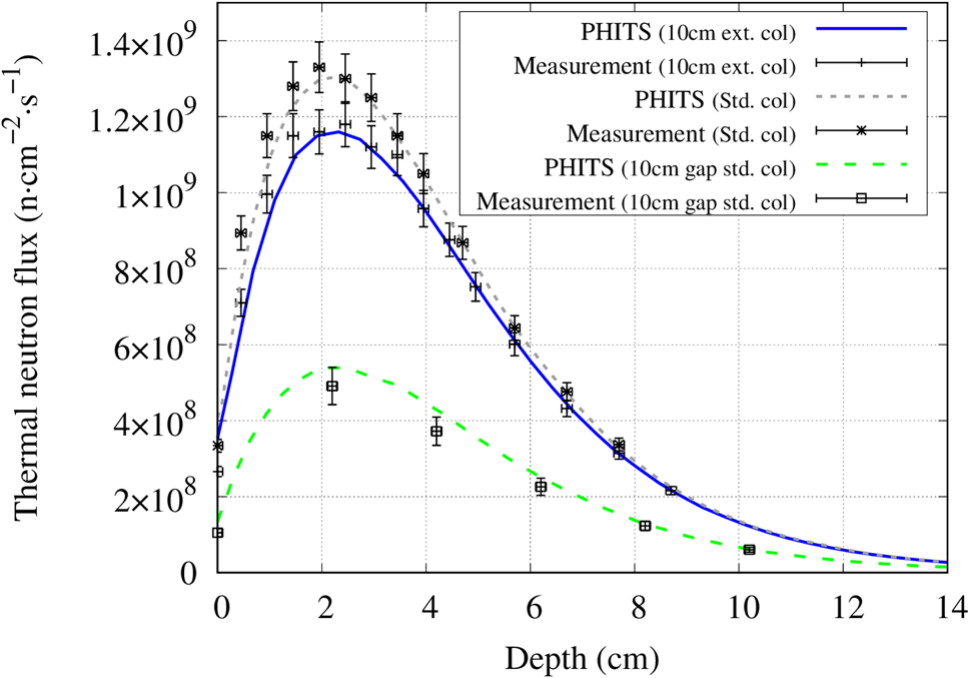
Central axis thermal neutron flux distribution inside a water phantom for the standard collimator, 10 cm extended collimator and the standard collimator with a 10 cm air gap between the collimator and the phantom.

The measured and simulated central axis thermal neutron flux distribution of the 5 cm and 10 cm extended collimator are shown in Figs. 5 and 6, respectively. The PHITS simulation agreed with the experimental measurements within the margin of the errors, expect for the surface region where the simulated values were larger than the experimental values. This was because the calculation uncertainty at the surface reduces due to the rapidly increasing thermal neutrons at this region. The same results were reported by Kumada et al.^15,16^.

**Figure 7:**
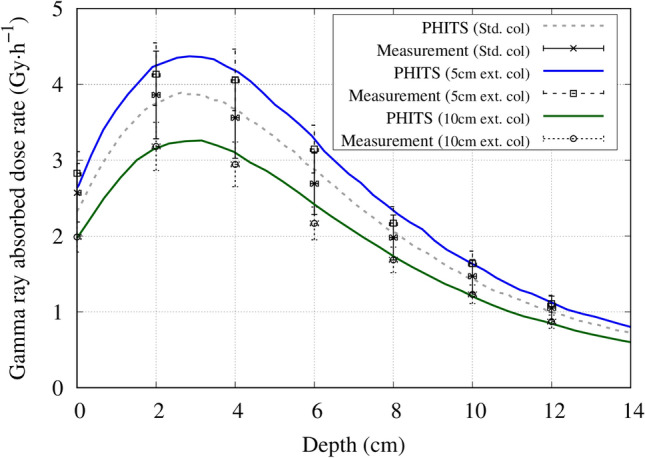
Central axis gamma ray dose distribution inside a water phantom for the standard collimator, 5 cm extended collimator and the 10 cm extended collimator.

**Figure 8:**
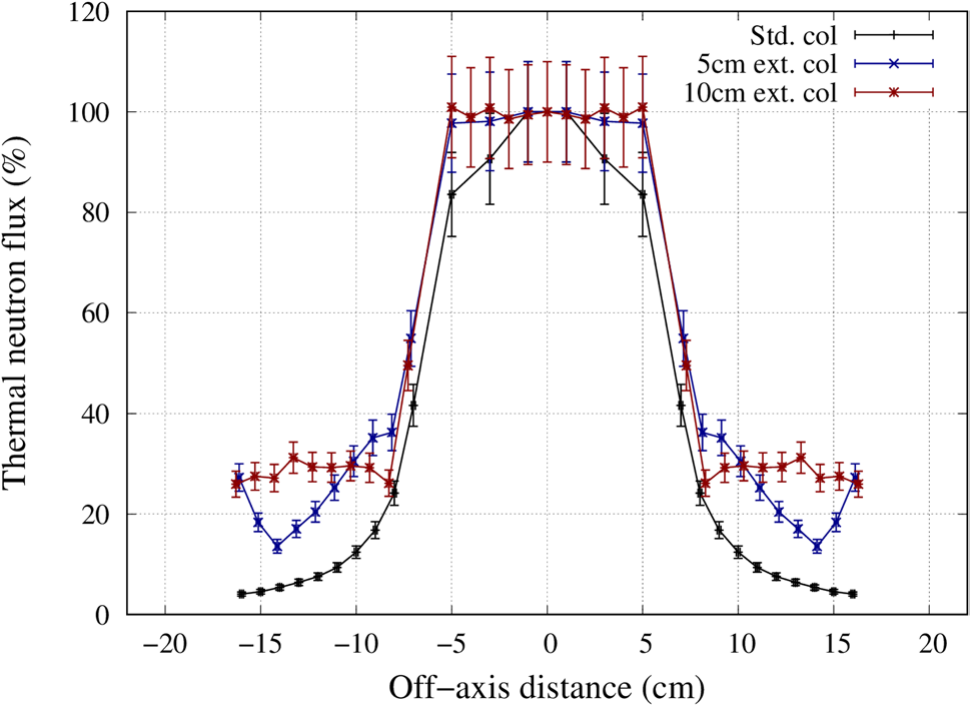
Thermal neutron flux distribution measured free-in-air along the collimator surface for the standard collimator, the 5 cm extended collimator and the 10 cm extended collimator.

**Figure 9:**
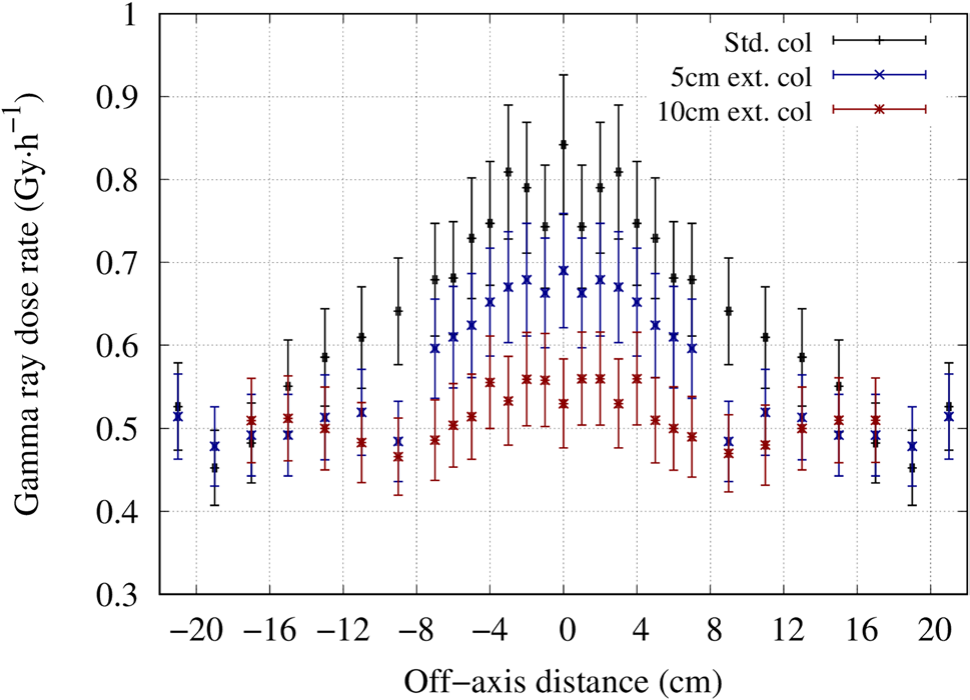
Gamma ray dose distribution measured free-in-air along the collimator surface for the standard collimator and the 5 cm extended collimator.

Under the same condition (source to skin distance is equal, where the source position is defined at the beam shaping assembly exit) the thermal neutron flux along the central beam axis approximately doubled with the extended collimator. The measured and simulated central axis gamma ray dose distribution for each collimator is shown in Fig. 7. The thermal neutron flux and gamma ray dose leakage from the collimators is shown in Figs. 8 and 9, respectively.

### Effect of an air gap

**Figure 10:**
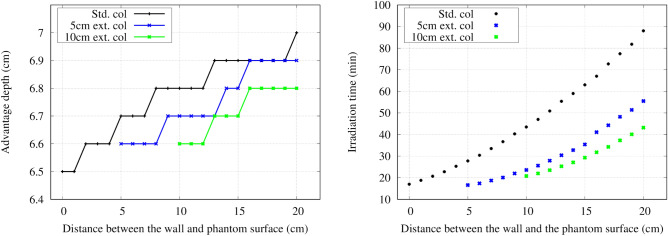
Left) Advantage depth as a function of the distance between the wall and the phantom surface for each collimator. Right) Irradiation time as a function of the distance between the wall and the phantom surface for each collimator.

**Figure 11:**
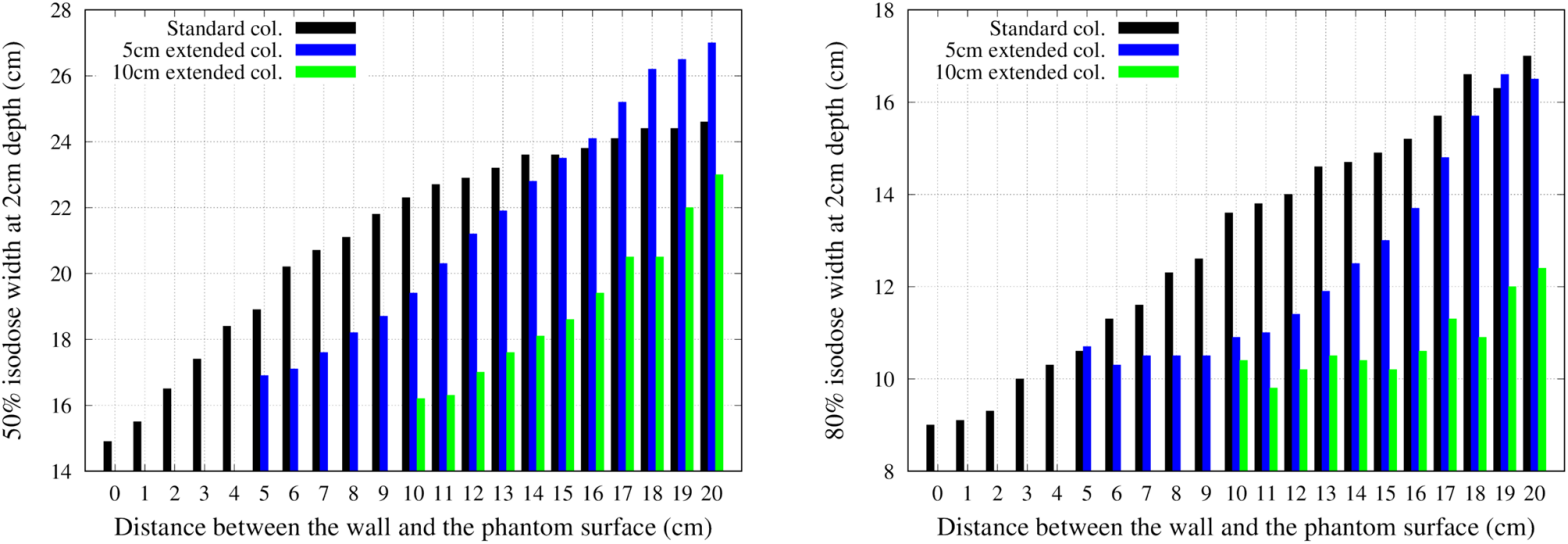
The change in the 50% (left) and 80% (right) isodose width at a depth of 2 cm with varying the distance between the wall and the phantom surface.

**Figure 12:**
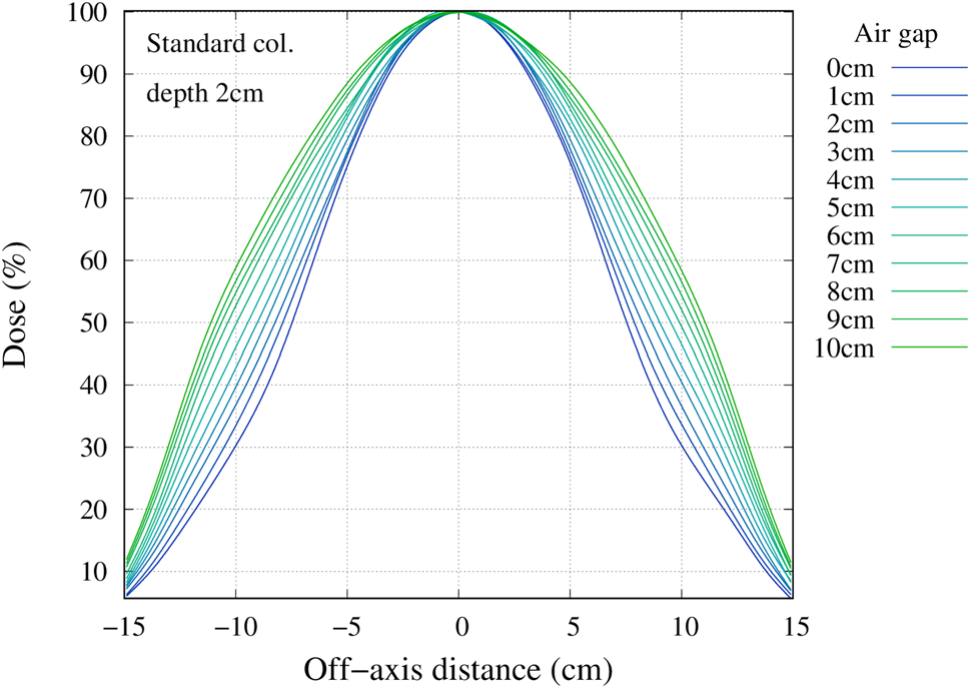
Off axis dose profile at a depth of 2 cm for the standard collimator.

**Figure 13:**
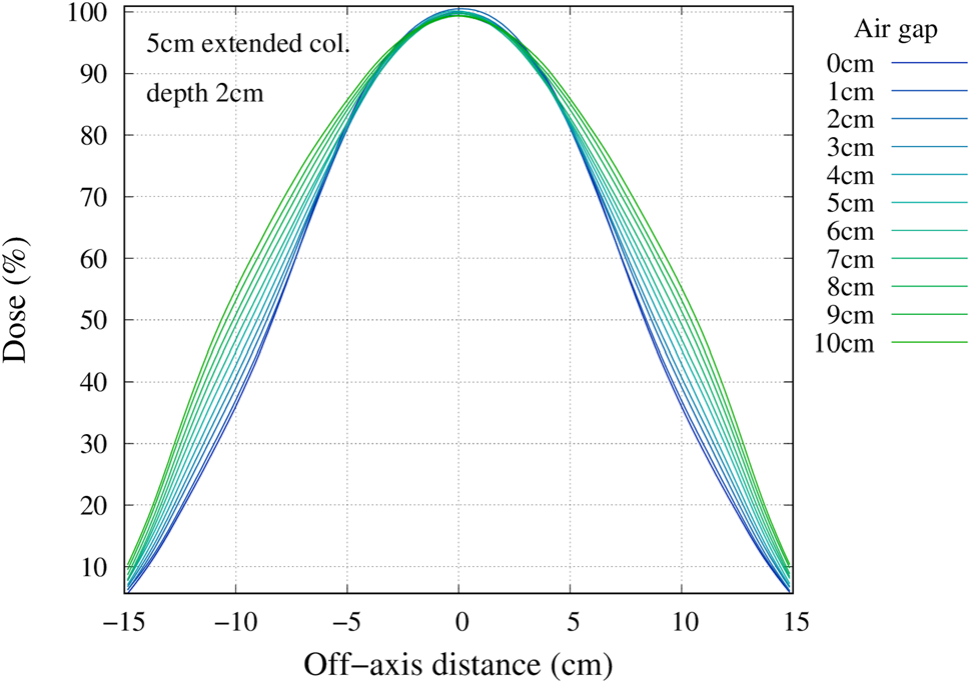
Off axis dose profile at a depth of 2 cm for the 5 cm extended collimator.

**Figure 14:**
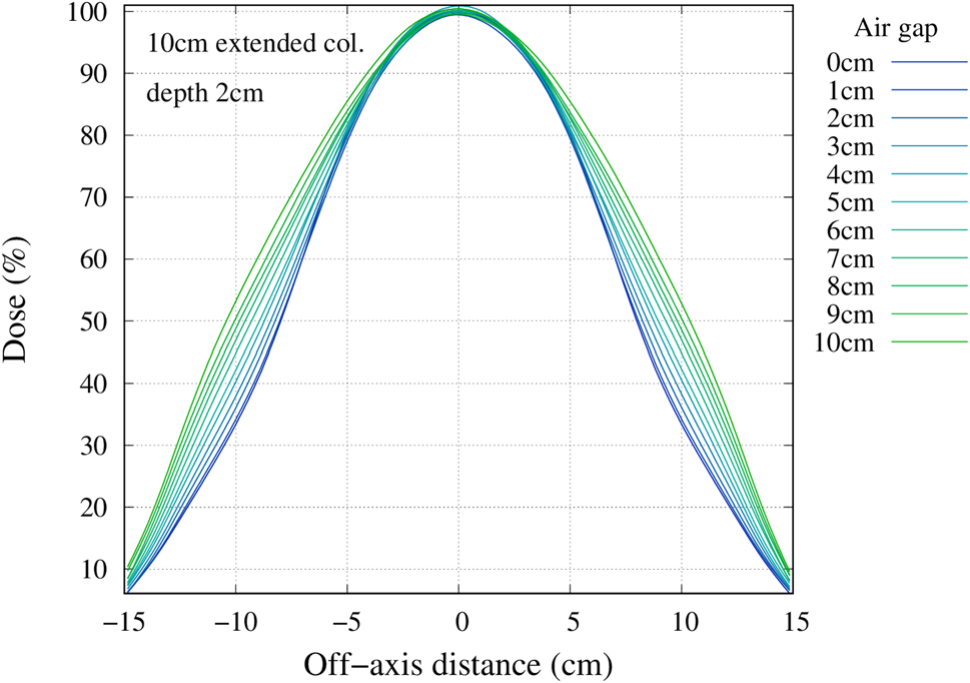
Off axis dose profile at a depth of 2 cm for the 10 cm extended collimator.

The advantage depth and irradiation time as a function of the distance between the wall and the phantom surface for the different collimators are shown in Fig. 10. The advantage depth marginally increased with the increase in the air gap. The irradiation time significantly reduced with the extended collimator. Figure 11 shows the change in the 80% and 50% isodose width at a depth of 2 cm with increasing air gap. The increase in the air gap increased the effective field size, as shown in the Figs. 12, 13, and 14, respectively.

### Treatment planning of a mock head and neck BNCT

#### Case 1

**Figure 15:**
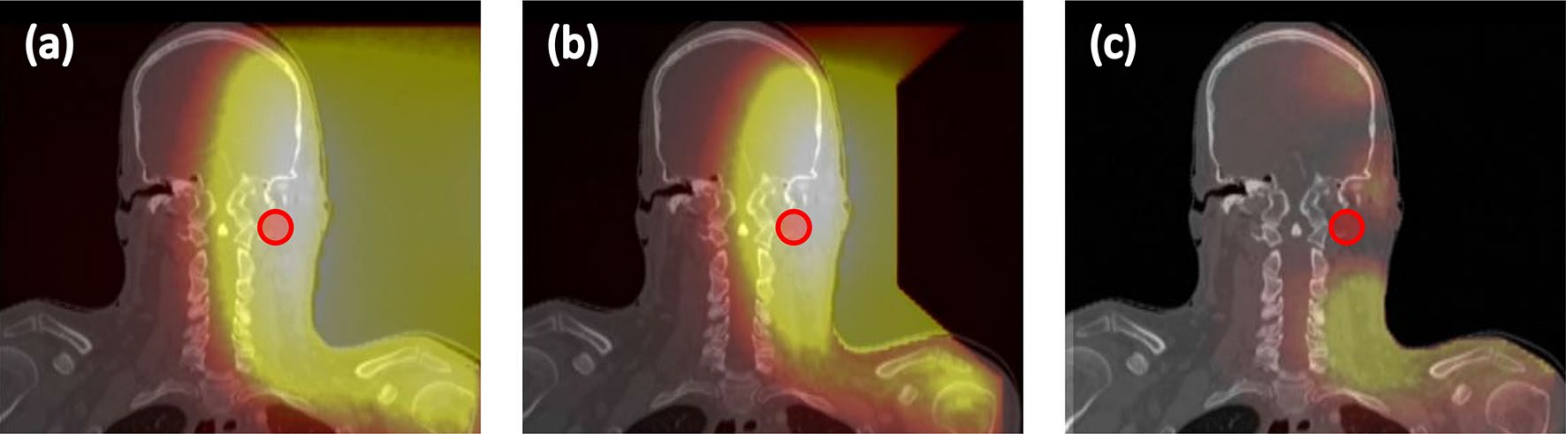
Graphical representation of the dose distribution of the (**a**) standard collimator, (**b**) 10 cm extended collimator, (**c**) dose difference for case 1.

**Figure 16:**
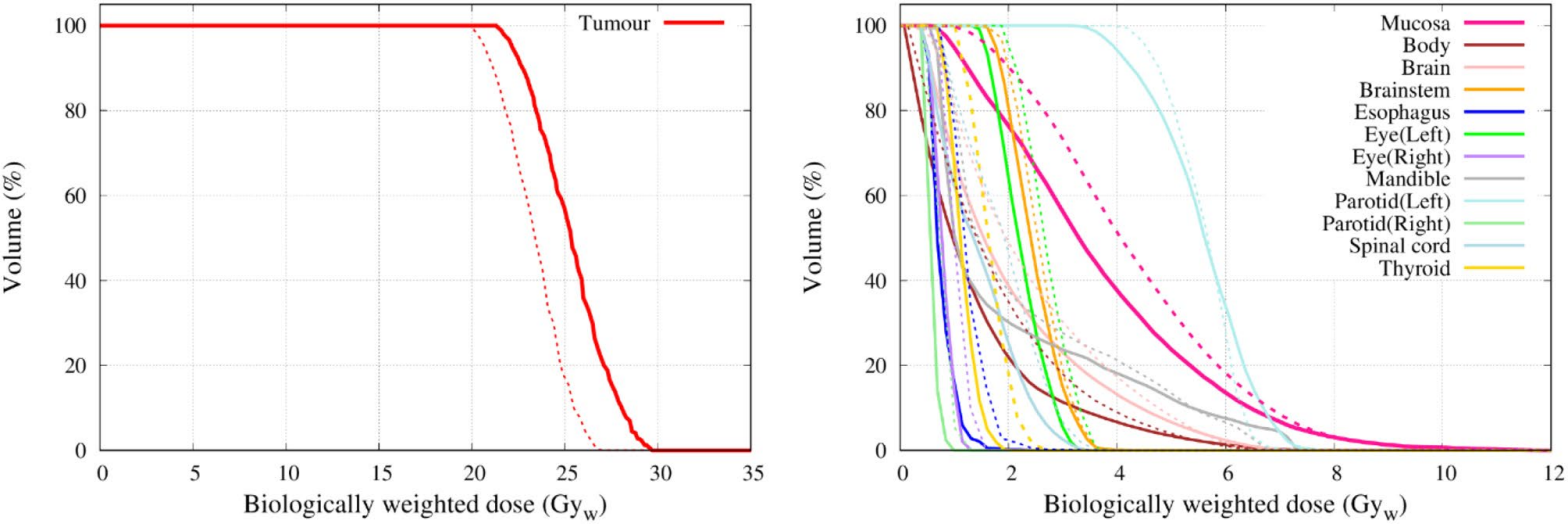
Dose volume histogram of the tumour (left) and the organs at risk (right) for case 1. The solid line is the dose distribution of the 10 cm extended collimator, and the dotted line is the dose distribution of the standard collimator.

Figure 15 shows the dose distribution for case 1 (left nasopharynx). The figures from left to right indicate the dose distribution (when the maximum dose of the mucosa membrane was set to 12 Gy_w_) for the standard collimator, the 10 cm extended collimator and the dose difference between the two collimators. The dose distribution of the standard collimator was more spread out, with the effect being more prominent near the neck and shoulder region. Table 2 shows the dose volume histogram parameters of the tumour and organs at risk and Fig. 16 shows the graph. The total irradiation time required to deliver a total dose of 12 Gy_w_ to the mucosa membrane for the standard collimator was calculated to be 86.5 min and 46.1 min with the 10 cm extended collimator.

**Table 2:** Summary of the DVH parameters for case 1 for both collimators.

Structure	Biologically weighted dose (Gy_w_)											
	D_min_		D_max_		D_1_		D_50_		D_95_		D_99_	
	Std	Ext	Std	Ext	Std	Ext	Std	Ext	Std	Ext	Std	Ext
Tumour	20.0	21.4	26.8	29.7	26.6	29.4	23.4	25.3	20.7	22.2	20.1	21.5
Mucosa	0.8	0.5	12.0	12.0	9.3	9.3	4.1	3.3	1.6	1.0	1.2	0.7
Body	0.1	0.1	15.3	17.4	6.0	6.3	1.4	1.0	0.3	0.1	0.2	0.1
Brain	0.4	0.3	6.9	7.6	6.2	6.5	2.0	1.5	0.6	0.5	0.5	0.4
Brainstem	1.7	1.6	6.0	6.2	3.6	3.6	2.5	2.4	1.9	1.8	1.7	1.6
Spinal cord	0.7	0.4	3.9	3.7	3.5	3.2	1.9	1.3	0.8	0.5	0.7	0.4
Eye_L	1.9	1.4	3.7	3.3	3.6	3.2	2.7	2.2	2.0	1.6	1.9	1.5
Eye_R	0.7	0.5	1.5	1.2	1.6	1.2	1.0	0.8	0.7	0.5	0.5	0.4
Esophagus	0.7	0.4	3.5	2.5	2.3	1.6	1.2	0.7	0.8	0.5	0.7	0.4
Parotid_L	4.0	3.2	7.2	7.8	6.8	7.3	5.7	5.6	4.5	3.9	4.2	3.5
Parotid_R	0.5	0.4	1.2	1.0	1.2	0.9	0.7	0.6	0.5	0.4	0.4	0.4
Thyroid	1.0	0.6	2.7	2.0	2.5	1.9	1.6	1.1	1.1	0.8	1.0	0.7
Mandible	0.7	0.5	6.9	7.6	6.7	7.3	1.4	1.0	0.8	0.7	0.7	0.6

#### Case 2

**Figure 17:**
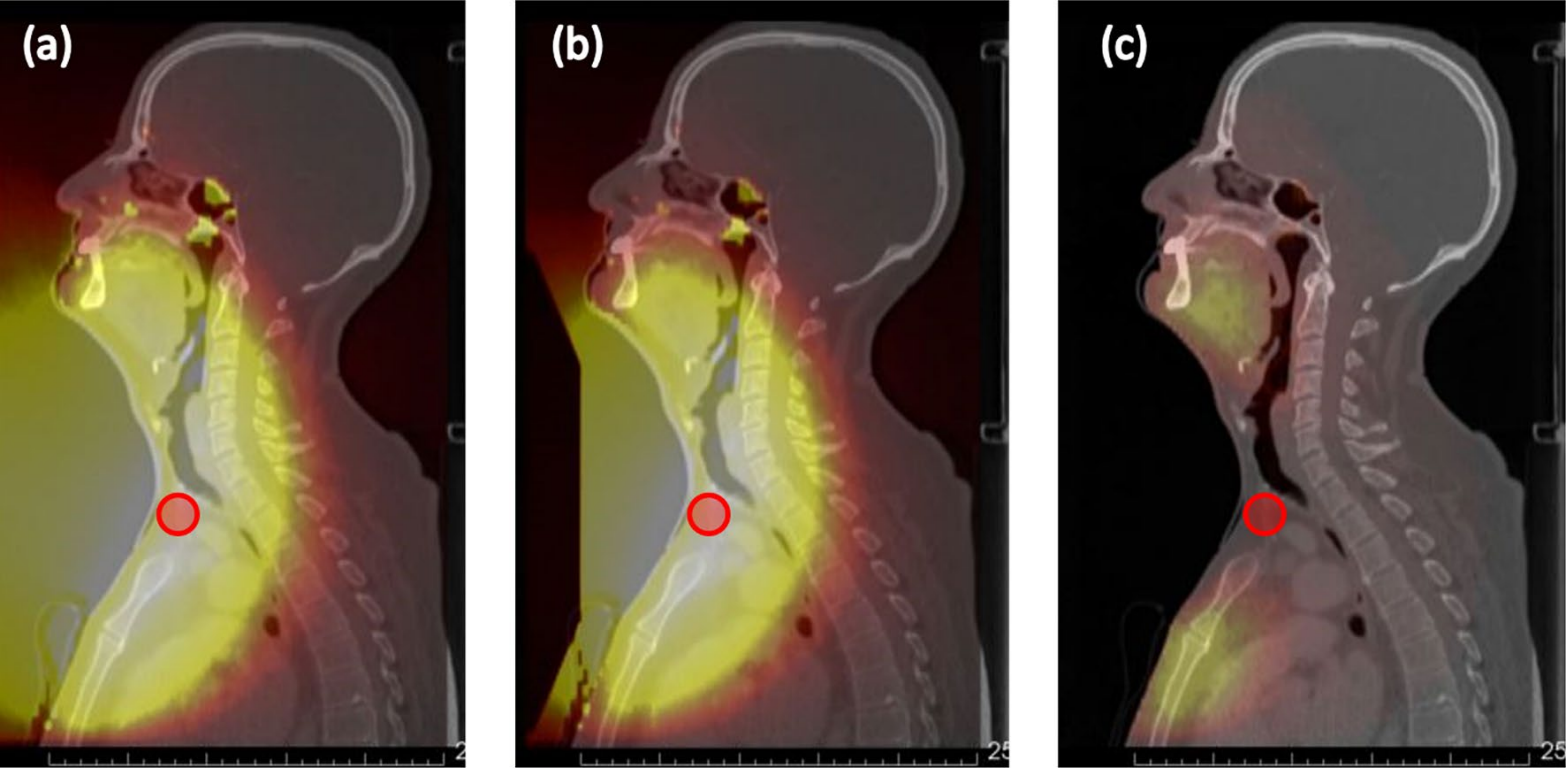
Graphical representation of the dose distribution of the (**a**) standard collimator, (**b**) 5 cm extended collimator, (**c**) dose difference for case 2.

**Figure 18:**
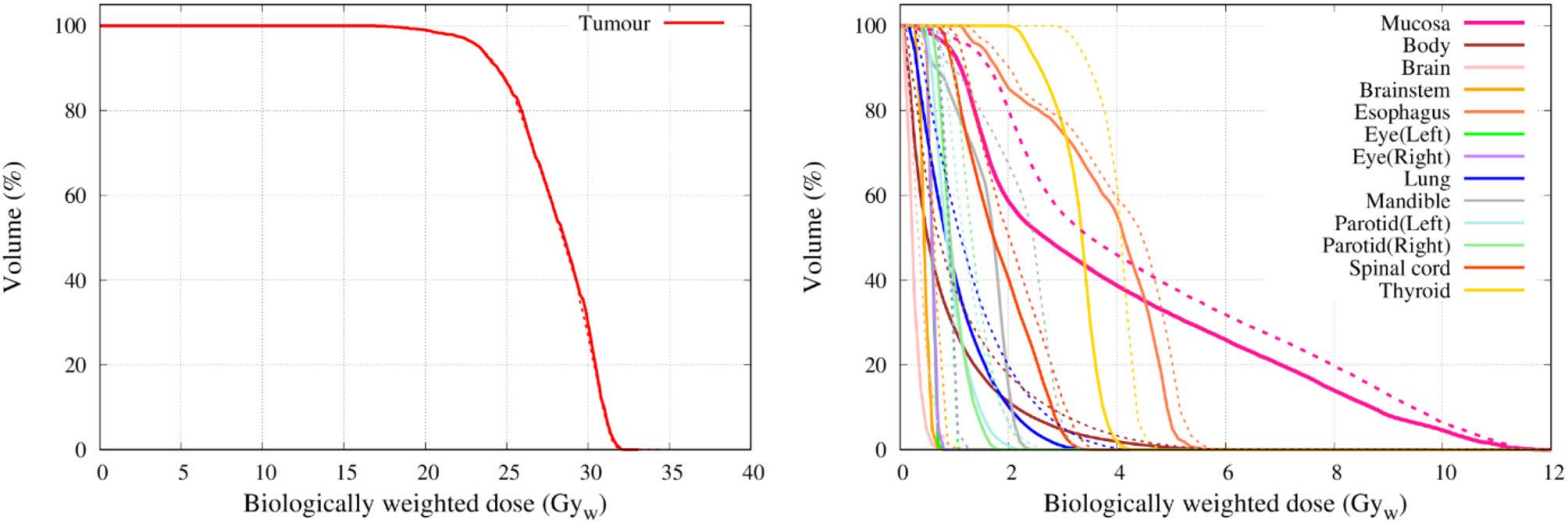
Dose volume histogram of the tumour (left) and the organs at risk (right) for case 2. The solid line is the dose distribution of the 10 cm extended collimator, and the dotted line is the dose distribution of the standard collimator.

Figure 17 shows the dose distribution for case 2 (hypopharynx). The figures from left to right indicate the dose distribution (when the maximum dose of the mucosa membrane was set to 12 Gy_w_) for the standard collimator, the 5 cm extended collimator and the dose difference between the two collimators. The dose distribution of the standard collimator is more spread out, with the effect being more prominent near the oral cavity and chest region. Table 3 shows the dose volume histogram parameters of the tumour and organs at risk and Fig. 18 shows the graph. The total irradiation time required to deliver a total dose of 12 Gy_w_ to the mucosa membrane for the standard collimator was calculated to be 58.5 min and 32.4 min with the 5 cm extended collimator.

**Table 3:** Summary of the DVH analysis for case 2 for both collimators.

Structure	Biologically weighted dose (Gy_w_)											
	D_min_		D_max_		D_1_		D_50_		D_95_		D_99_	
	Std	Ext	Std	Ext	Std	Ext	Std	Ext	Std	Ext	Std	Ext
Tumour	16.2	16.1	31.9	32.2	31.7	31.8	28.4	28.5	23.3	23.3	20.1	20.1
Mucosa	0.3	0.2	12.0	12.0	11.3	11.0	3.5	2.7	1.3	0.9	0.6	0.4
Lung	0.2	0.1	4.5	3.7	3.7	3.0	1.1	0.8	0.4	0.3	0.3	0.2
Body	0.1	0.1	18.8	18.7	5.0	4.6	0.7	0.5	0.2	0.1	0.1	0.1
Brain	0.1	0.1	1.6	1.1	0.9	0.6	0.3	0.2	0.1	0.1	0.1	0.1
Brainstem	0.4	0.3	1.0	0.7	1.0	0.7	0.6	0.4	0.4	0.3	0.3	0.3
Spinal cord	0.7	0.5	3.7	3.5	3.5	3.2	2.0	1.7	1.1	0.9	0.9	0.8
Eye_L	0.7	0.4	1.1	0.7	1.1	0.7	0.9	0.6	0.7	0.5	0.7	0.4
Eye_R	0.7	0.4	1.1	0.8	1.2	0.8	0.9	0.6	0.7	0.5	0.7	0.4
Esophagus	1.3	1.2	6.4	6.2	5.6	5.3	4.5	4.1	1.7	1.5	1.3	1.2
Parotid_L	0.7	0.5	2.7	2.2	2.4	2.0	1.2	0.9	0.7	0.5	0.7	0.5
Parotid_R	0.8	0.6	2.4	1.8	2.2	1.7	1.3	0.9	0.9	0.6	0.9	0.6
Thyroid	2.9	2.0	4.9	4.2	4.7	4.1	4.1	3.4	3.3	2.4	3.1	2.2
Mandible	0.6	0.4	3.4	2.4	3.2	2.3	2.5	1.7	0.7	0.5	0.6	0.4

In both cases, when the dose was normalised to the mucosal membrane and the source to skin distance was kept the same, the irradiation time was significantly shorter with the extended collimator when compared to the standard collimator. The dose to the organs outside the irradiation field were reduced and the dose delivered to the tumour was improved for case 1.

## Discussion

The experimental measurements of the thermal neutron flux and the gamma ray dose rate inside the water phantom closely matched the simulation results. By extending the collimator, the distance from the BSA to the collimator exit was increased, which decreased the neutron intensity as neutrons scatter in air. However, the decrease in the neutron intensity was compensated by increasing the volume inside the collimator. The neutron leakage rate (per second) measured at the surface of the collimator was found to be higher for the extended collimator when compared with the standard collimator. However, the irradiation time was significantly reduced with the extended collimator (under the same condition). Therefore, when the irradiation time was normalised to the mucosal membrane (time required to deliver 12 Gy_w_ to the mucosal membrane), the total number of neutrons leaking from the collimator was found to be less than or approximately the same as the standard collimator. Also, the spread of neutron beam was reduced with the extended collimator in comparison with the standard collimator with an air gap present. Overall, the use of the extended collimator reduced the out-of-field dose. Unlike conventional X-ray or proton therapy, where the particles mostly travel in a straight line before reaching the patient, epithermal neutrons scatter in air before reaching the patient, resulting in a gaussian like shaped dose profile. Therefore, the physical collimator size (12 cm diameter for this study) does not indicate the dose profile width inside the patient. The profile width changes with the air gap and the depth inside the patient. So, care must be taken when selecting the collimator size to make sure the target is covered by the desired isodose line. In this study, a fixed RBE/CBE factor was used to determine the total weighted dose. There are other calculation models (for e.g. the photon-isoeffective dose^17^), which may produce a different total dose distribution in contrast to the fixed RBE/CBE method.

For most head and neck cases, due to the patient anatomy, an air gap between the patient and the collimator surface exists. For a case where there is no air gap present, the use of the standard collimator may be preferable as there is almost no difference in the treatment time and the leakage from the collimator was found to be lower. This was shown from the in-air measurement of the off-axis neutron profile measured for the different collimators. The profile for the extended collimator was flatter when compared with the standard collimator. The shape of the standard collimator was tapered then straightened out, where the extended collimator was designed to be tapered all the way to the beam exit. The effect of straightening out the downstream of the collimator attenuated the neutron beam near the edges, producing a curved, gaussian-shaped beam profile. The beam profile was also narrower with the standard collimator, indicating for the same irradiation time, the neutron leakage was less with the standard collimator. Also, there were increases in the thermal neutron leakage beyond 14 cm from the center. This was due to the slight air gap present when the extended collimator was attached to the existing collimator. The slight increase in the thermal neutron leakage between 12 and 14 cm for the 10 cm extended collimator was due to the holes drilled out for the screws to attach the collimator material together. These leakages can be reduced by using a tongue and groove design and patching up the holes with an attenuating material.

For a slightly deep-seated tumour (5–6 cm), the use of the extended collimator increased the tumour dose by approximately 7%. The advantage depth marginally increased with increasing air gap. No significant difference in the tumour dose between the extended collimator and the standard collimator was observed for a shallow tumour. For the organs at risk, a significant reduction was observed with the use of the extended collimator, particularly for the mucosal membrane (approximately 25% reduction at D_50_), which is usually the organ where the dose is prescribed to for head and neck BNCT. The treatment time was significantly reduced (approximately up to 60% reduction) with the use of the extended collimators. This is important not only from the patient comfort point of view, but it may also reduce the patient motion during treatment. However, for case 1, it was found the maximum dose of the brain, brainstem, and left parotid slightly increased with the extended collimator. This may be due to the increase in the thermal neutron distribution, particularly along the central beam axis, as the direction of neutrons become more forward directed with the use of the extended collimator. Although the dose to the tumour increased, the dose to the OAR’s near the center of the field also increased and this needs to be considered when generating the treatment plan.

## Conclusion

The 5 cm and 10 cm extended collimators for clinical BNCT application were designed, manufactured and experimental measurements confirmed the simulation results. The extended collimators significantly reduced the irradiation time (when the dose was normalised to the mucosal membrane), and the treatment would be performed in a much comfortable position. For BNCT, the dose profile width inside the patient is not determined by the physical collimator size. The profile width varies depending on the collimator shape, air gap, and depth inside the patient. Therefore, when selecting the collimator size, it is important to ensure the target is covered by the specified isodose line. The simulation results showed the dose delivered to the organs at risk out of field were significantly reduced with the use of the extended collimators and the dose delivered to deep-seated tumours slightly increased for the same prescription dose of 12 Gy-eq to the mucosal membrane. The application of the extended collimators may increase the indication of head and neck BNCT.

## Data Availability

The datasets generated during and/or analysed during the current study are available from the corresponding author on reasonable request.

## References

[1] Kato I (2004). Effectiveness of BNCT for recurrent head and neck malignancies. Appl. Radiat. Isot..

[2] Aihara T (2014). Boron neutron capture therapy for advanced salivary gland carcinoma in head and neck. Int. J. Clin. Oncol..

[3] Kankaanranta L (2012). Boron neutron capture therapy in the treatment of locally recurred head-and-neck cancer: final analysis of a phase I/II trial. Int. J. Radiat. Oncol..

[4] Haapaniemi A (2016). Boron neutron capture therapy in the treatment of recurrent laryngeal cancer. Int. J. Radiat. Oncol..

[5] Tanaka, H. *et al.* Characteristics comparison between a cyclotron-based neutron source and KUR-HWNIF for boron neutron capture therapy. *Nucl. Instrum. Methods Phys. Res. Sect. B Beam Interact. Mater. Atoms* **267**, 1970–1977 (2009).

[6] Tanaka H (2011). Experimental verification of beam characteristics for cyclotron-based epithermal neutron source (C-BENS). Appl. Radiat. Isot..

[7] Hirose K (2021). Boron neutron capture therapy using cyclotron-based epithermal neutron source and borofalan (10B) for recurrent or locally advanced head and neck cancer (JHN002): an open-label phase II trial. Radiother. Oncol..

[8] Sato, T. *et al.* Features of particle and heavy ion transport code system (PHITS) version 3.02. *J. Nucl. Sci. Technol.* **55**, 684–690 (2018).

[9] Sakurai, Y. & Kobayashi, T. Characteristics of the KUR Heavy Water Neutron Irradiation Facility as a neutron irradiation field with variable energy spectra. *Nucl. Instrum. Methods Phys. Res. Sect. A Accel. Spectrometers Detect. Assoc. Equip.* **453**, 569–596 (2000).

[10] Hu N (2021). Evaluation of a treatment planning system developed for clinical boron neutron capture therapy and validation against an independent Monte Carlo dose calculation system. Radiat. Oncol..

[11] Coderre JA (1993). Derivations of relative biological effectiveness for the high-let radiations produced during boron neutron capture irradiations of the 9l rat gliosarcoma in vitro and in vivo. Int. J. Radiat. Oncol. Biol. Phys..

[12] Coderre JA (1998). Biodistribution of boronophenylalanine in patients with glioblastoma multiforme: boron concentration correlates with tumor cellularity. Radiat. Res..

[13] Fukuda H (2003). Boron neutron capture therapy (BNCT) for malignant melanoma with special reference to absorbed doses to the normal skin and tumor. Australas. Phys. Eng. Sci. Med..

[14] Coderre JA, Morris GM, Micca PL, Fisher CD, Ross GA (1995). Comparative assessment of single-dose and fractionated boron neutron capture therapy. Radiat. Res..

[15] Kumada H (2004). Verification of the computational dosimetry system in JAERI (JCDS) for boron neutron capture therapy. Phys. Med. Biol..

[16] Kumada H (2020). Verification for dose estimation performance of a Monte-Carlo based treatment planning system in University of Tsukuba. Appl. Radiat. Isot..

[17] González SJ, Santa Cruz GA (2012). The photon-isoeffective dose in Boron neutron capture therapy. Radiat. Res..

